# Associations Between Serum IL‐17A, Renal Function and Diabetic Retinopathy in Type 2 Diabetes Mellitus: Evidence From a Chinese Han Population

**DOI:** 10.1002/edm2.70033

**Published:** 2025-02-13

**Authors:** Wensu Wang, Yan Huang, Jianguo Shen, Li Jin, Zhuo Chen

**Affiliations:** ^1^ Department of Geriatrics The Second Affiliated Hospital of Guizhou University of TCM Guiyang Guizhou China; ^2^ Division of Nephrology Affiliated Hospital of Chengde Medical College Chengde China; ^3^ Department of Endocrinology and Metabolism First Affiliated Hospital, School of Medicine, Zhejiang University Hangzhou Zhejiang China; ^4^ Shanghai Diabetes Institute, Shanghai Key Laboratory of Diabetes Mellitus, Shanghai Clinical Center for Diabetes Shanghai Jiaotong Universuty Affiliated 6th People's Hospital Shanghai China

## Abstract

**Background:**

Evidence suggested that IL‐17A was associated with renal function in type 2 diabetes. We used ultra‐sensitive measurement to detect the concentration of IL‐17A in human peripheral blood and explored the association of IL‐17A with diabetic kidney disease (DKD).

**Methods:**

We recruited 138 participants from the Shanghai Diabetes Institute Inpatient Database of Shanghai Jiao Tong University Affiliated Sixth People's Hospital. Eighty‐four individuals diagnosed as DKD were cases, and 54 type 2 diabetes individuals without DKD or diabetic retinopathy (DR) were controls. The concentration of serum IL‐17A was detected by the High Sensitivity Immunoassay Quantitative Kit. Data was analysed by SAS.

**Results:**

The concentration of serum IL‐17A in our population ranged from 0.07 pg/mL to 2.96 pg/mL with the median of 0.502 pg/mL. Our results suggested that the level of serum IL‐17A in DKD case group was higher than in the control (*P*_*unadjusted*_ = 0.0496, *P*_*adjusted*_ = 0.0298). And serum creatinine, eGFR, ACR were used as indicators of renal function. Serum creatinine and ACR were positive correlated with the level of serum IL‐17A (*P*_*adjusted*_ = 0.0148; *P*_*adjusted*_ = 0.0369), while eGFR showed a negative correlation (*P*_*adjusted*_ = 0.0167). Additionally, the level of serum IL‐17A was also significantly higher in DR case group compared with the control group (*p* = 0.0224).

**Conclusion:**

Serum IL‐17A level is associated with renal function decline and diabetic retinopathy in patients with type 2 diabetes in a Chinese Han population. Our results suggested that IL‐17A may be a potential biomarker of DKD and DR.

## Graphical abstract

This study investigates the relationship between serum IL‐17A levels and diabetic kidney disease (DKD) in individuals with type 2 diabetes. Using ultra‐sensitive assays, we measured IL‐17A concentrations in 138 participants, including 84 with DKD and 54 without complications. Results showed higher serum IL‐17A levels in the DKD group compared to controls, with a significant correlation between IL‐17A and markers of renal function, including serum creatinine and albumin‐to‐creatinine ratio (ACR), and an inverse correlation with estimated glomerular filtration rate (eGFR). Additionally, IL‐17A was elevated in individuals with diabetic retinopathy (DR). These findings suggest IL‐17A as a potential biomarker for both DKD and DR in type 2 diabetes, highlighting its role in renal function decline.

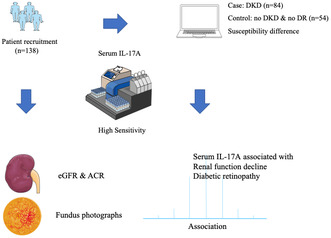

## 1 Introduction

With the growing epidemic of diabetes in China, diabetic kidney disease (DKD) has become a major health concern, exerting a profound impact on the Chinese population. Nowadays, it is estimated that more than 100 million individuals have diabetes in China [1], and approximately 40% of those with type 2 diabetes mellitus (T2DM) will develop DKD [2]. As the leading cause of chronic kidney disease and end‐stage renal disease, DKD resulted in over 80,000 deaths in China in 2016 alone [3]. Furthermore, patients with DKD are at significantly higher risk of developing cardiovascular diseases compared to those with diabetes but without DKD [4, 5]. Despite advancements in diabetes management, there are still no effective measures to halt the progression of established DKD. By the time serum creatinine levels decrease, more than half of kidney function is already lost. Early intervention can effectively slow DKD progression [6, 7], but even intensive therapy cannot reverse its course [8, 9, 10]. This underscores the urgent need for sensitive biomarkers to identify DKD at an early stage and for novel therapeutic targets to combat its progression.

Diabetic microvascular complications, including DKD and diabetic retinopathy (DR), are the primary contributors to morbidity and mortality in patients with T2DM. DR, another major microvascular complication of diabetes, is the leading cause of blindness in working‐age adults worldwide [11]. DR and DKD often coexist, reflecting their shared pathophysiological mechanisms such as chronic hyperglycemia, inflammation and vascular dysfunction. Studies have shown that patients with DKD are more likely to develop DR, and the severity of DR often parallels the progression of DKD [12]. These microvascular complications significantly impact patients' quality of life and impose a substantial socioeconomic burden. A deeper understanding of the molecular pathways driving these complications could aid in developing new interventions to mitigate their progression.

Inflammation plays a pivotal role in the pathogenesis of diabetes and its microvascular complications. Among various inflammatory mediators, interleukin‐17A (IL‐17A) has gained increasing attention due to its critical role in inflammatory processes. IL‐17A is a cytokine predominantly secreted by CD4+ T cells, and it is also produced by other immune cells, such as natural killer cells, neutrophils, macrophages, dendritic cells, lymphoid tissue inducer cells, mast cells and plasma cells [13, 14]. Nowadays, IL‐17A get more and more attention because of its important role in the inflammatory process. A key function of IL‐17A is its ability to induce chemokines, including CXCL1, CXCL2 and CXCL8, thereby attracting neutrophils to sites of infection or injury [15, 16]. Elevated serum IL‐17A levels have been observed in a variety of inflammatory and metabolic diseases, including psoriasis, oral lichen planus and systemic lupus erythematosus [17, 18, 19]. This makes IL‐17A a potentially sensitive marker of inflammation. [17, 18, 20].

Even though the pleiotropic functions of IL‐17A in inflammation are still being actively investigated, accumulating evidence indicates its critical role in DKD [19, 21]. In a mouse model of diabetes, mycophenolate mofetil attenuated diabetic nephropathy by reducing T lymphocyte infiltration in the kidneys, with an increase in IL‐17A+ CD4+ T cells observed as diabetic nephropathy progressed [22, 23]. Furthermore, recent studies have demonstrated that IL‐17A blockade can significantly reduce albuminuria and kidney injury in mouse models of diabetic nephropathy, reinforcing its potential as a therapeutic target [24]. Interestingly, early research reported conflicting findings. For instance, a study published in 2016 found that IL‐17A knockout exerted a protective effect against the development of DKD, while low‐dose IL‐17 therapy was shown to reduce albuminuria [25]. This paradoxical role of IL‐17A highlights the complexity of its function in DKD and underscores the need for further research to clarify its dualistic effects [26]. These findings collectively suggest that while IL‐17A plays an essential role in DKD pathogenesis, its precise mechanisms and therapeutic potential require deeper investigation.

Given the overlapping pathways between DKD and DR, it is crucial to investigate whether IL‐17A also plays a dual role in the development of DR. While significant progress has been made in understanding the role of IL‐17A in DKD, its association with DR in patients with T2DM remains largely unexplored. Given these findings, it is important to explore the differences in serum IL‐17A levels between patients with DKD and those with T2DM but without DKD. However, measuring serum IL‐17A levels in diabetes patients presents significant challenges, as its concentrations are typically very low—often just a few pg/mL—and are difficult to detect with conventional methods. Previous commercial ELISA kits for serum IL‐17A detection had detection thresholds that were too high to reliably measure IL‐17A levels in diabetes or DKD patients. The advent of the Human IL‐17A High Sensitivity Immunoassay Kit provides an advanced tool to address this limitation, enabling the detection of serum IL‐17A at lower levels.

In this study, we leveraged this ultra‐sensitive detection method to investigate the relationship between serum IL‐17A levels and DKD. Our aim was to identify whether IL‐17A could serve as a sensitive biomarker for DKD or provide evidence supporting its potential as a therapeutic target for this severe complication.

## 2 Methods

### 2.1 Ethnical Approval

Ethical approval of our study was granted by the institutional review board of Shanghai Jiao Tong University Affiliated Sixth People's Hospital according to the principles of the Helsinki Declaration II. Informed consents were obtained from each participant.

### 2.2 Participants

One hundred and thirty‐eight participants older than 18 years old from the Shanghai Diabetes Institute Inpatient Database of Shanghai Jiao Tong University Affiliated Sixth People's Hospital were recruited for this research. All the patients had Chinese Han ancestry and were diagnosed with T2DM according to the 1999 WHO criteria (fasting plasma glucose ≥ 7.0 mmol/L and/or 2 h plasma glucose ≥ 11.1 mmol/L). Type 1 diabetes, mitochondrial diabetes, other specific types of diabetes, other types of kidney disease, cancers, severe mental disorders and disabilities were excluded. Of the 138 T2DM patients, 84 individuals diagnosed as DKD (albumin‐to‐creatinine ratio > 30 𝜇g/mg or eGFR < 60 mL/min per 1.73m^2^) were DKD cases, and 54 whose renal function were normal (albumin‐to‐creatinine ratio < 30 𝜇g/mg and eGFR > 90 mL/min per 1.73m^2^) without diabetic retinopathy (DR) were controls.

### 2.3 Clinical Phenotypes

The clinical data such as sex, age, BMI, duration of diabetes, HbA1c, serum creatinine, serum uric acid, blood urea nitrogen, microalbuminuria was collected. Body mass index (BMI) was calculated as weight(kg)/height^2^(m^2^). HbA1c levels were detected with high‐performance liquid chromatography (HPLC) by the Bio‐Rad Variant II Haemoglobin Testing System (Bio‐Rad Laboratories, Hercules, CA, USA). Microalbuminuria was recoded as ACR (albumin‐to‐creatinine ratio). ACR were measured in three different days, and the mean of them was used for analysis. ACR was measured with scatter turbidimetry by the BN II System (Siemens Healthcare Diagnostics Products GmbH, Marburg, Germany). The modified MDRD equation for Chinese was used to calculate eGFR [27]. Fundus photographs of individuals were conducted using a 45∘ 6.3 megapixel digital nonmydriatic camera (Canon CR6‐45NM; Lake Success, NY, USA). In this section, we followed the methods as we used before [28].

### 2.4 Determination the Concentration of IL‐17A

Blood samples were collected after 12‐h overnight fasting. Then centrifuged blood samples after resting to get serum and stored serum at −80°C until being detected. The IL‐17A concentration was detected in serum using the SMC Human IL‐17A High Sensitivity Immunoassay Kit for the Quantitative Determination of IL‐17A in Human Plasma and Serum (Catalogue # 03–0159‐00) according to manufacturer's instructions.

The lower limit of detection in this assay was 0.07 pg/mL. As the instruction reported, the mean intra‐assay variation was less than 10% and the mean inter‐assay variation was less than 15%. And the average spike recovery rate was 89%.

### 2.5 Statistical Analysis

The distributions of measurement data were conducted by the Normality test. The skewed distribution traits were compared by Wilcoxon Rank‐Sum test using SAS program (version 9.3; SAS Institute Cary, America). Composition ratio difference was performed with the chi‐square test. Rank correlation was used to test the relationship between the concentration of serum IL‐17A and other clinical phenotypes. GraphPad Prism 7.0 (GraphPad Software Inc., LaJolla, CA) was used to make figures. A two‐tailed *p*‐value of < 0.05 was considered significant.

## 3 Results

### 3.1 The Level of Serum IL‐17A in Patients With Type 2 Diabetes

A total number of 138 patients with T2DM were included in our research with the median age of 58.1 years old. The clinical characteristics of patients were summarised in Table 1. Of the 138 individuals, 84 individuals were cases and 54 individuals were controls. Compared with control group, the level of serum creatinine as well as albuminuria were higher and eGFR was lower in case group (*p* < 0.0001). What's more, the level of serum uric acid and blood urea nitrogen was lower in case group compared with control group (*p* < 0.05). Additionally, other characteristics including gender composition ratio, age, BMI, duration of T2DM and HbA1c had no significant difference between two groups.

**TABLE 1:** The basic characteristics of the case and control groups.

	Overall	Case	Control	*p*
Male/Female	91/47	56/28	35/19	0.8227
Age (year)	58.1 (50.8, 64.5)	59.7 (51.7, 65.6)	57.3 (50.5, 62.0)	0.1113
BMI (kg/m^2^)	24.8 (23.0, 28.3)	24.8 (23.4, 29.0)	24.7 (23.0, 27.1)	0.3550
Duration of diabetes (year)	10.0 (10.0, 14.0)	10.0 (6.0, 15.5)	10.0 (10.0, 12.0)	0.7141
Serum creatinine (umol/L)	63.0 (52.0, 81.0)	73.5 (58.0, 100.5)	53.0 (46.0, 65.0)	< 0.0001*
eGFR (mL/min/1.73m^2^)	138.5 (104.5, 173.6)	115.8 (90.0, 153.0)	159.2 (138.8, 193.4)	< 0.0001*
HbA1c (%)	9.3 (8.0, 10.2)	9.2 (7.8, 10.2)	9.4 (8.3, 10.2)	0.2033
ACR (μg/mg)	51.9 (2.1, 182.2)	128.1 (68.7, 314.0)	1.4 (0.8, 4.8)	< 0.0001*
Serum uric acid (umol/L)	304.0 (255.0, 372.0)	290.5 (224.0, 354.0)	317.0 (265.0, 399.0)	0.0370*
Blood urea nitrogen (mmol/L)	5.4 (4.4, 6.6)	5.1 (4.3, 6.2)	5.5 (4.4, 7.5)	0.0251*

*Note:* The data are summarised as median (interquartile range) for continuous variables. *p* values were calculated by Wilcoxon Rank‐Sum tests or chi‐square test. *p* values < 0.05 are shown in bold.

Abbreviations: ACR, albumin‐to‐creatinine ratio; BMI, body mass index; eGFR, estimated glomerular filtration rate.

*p < 0.05.

In patients with type 2 diabetes, the distribution of serum IL‐17A concentration was skewed (*p* < 0.0001). And the concentration of serum IL‐17A ranged from 0.07 pg/mL to 2.96 pg/mL with the median of 0.502 pg/mL. In addition, the interquartile range was 0.306 pg/mL to 0.832 pg/mL.

### 3.2 Patients With DKD Have Higher Level of Serum IL‐17A Than Control

**FIGURE 1:**
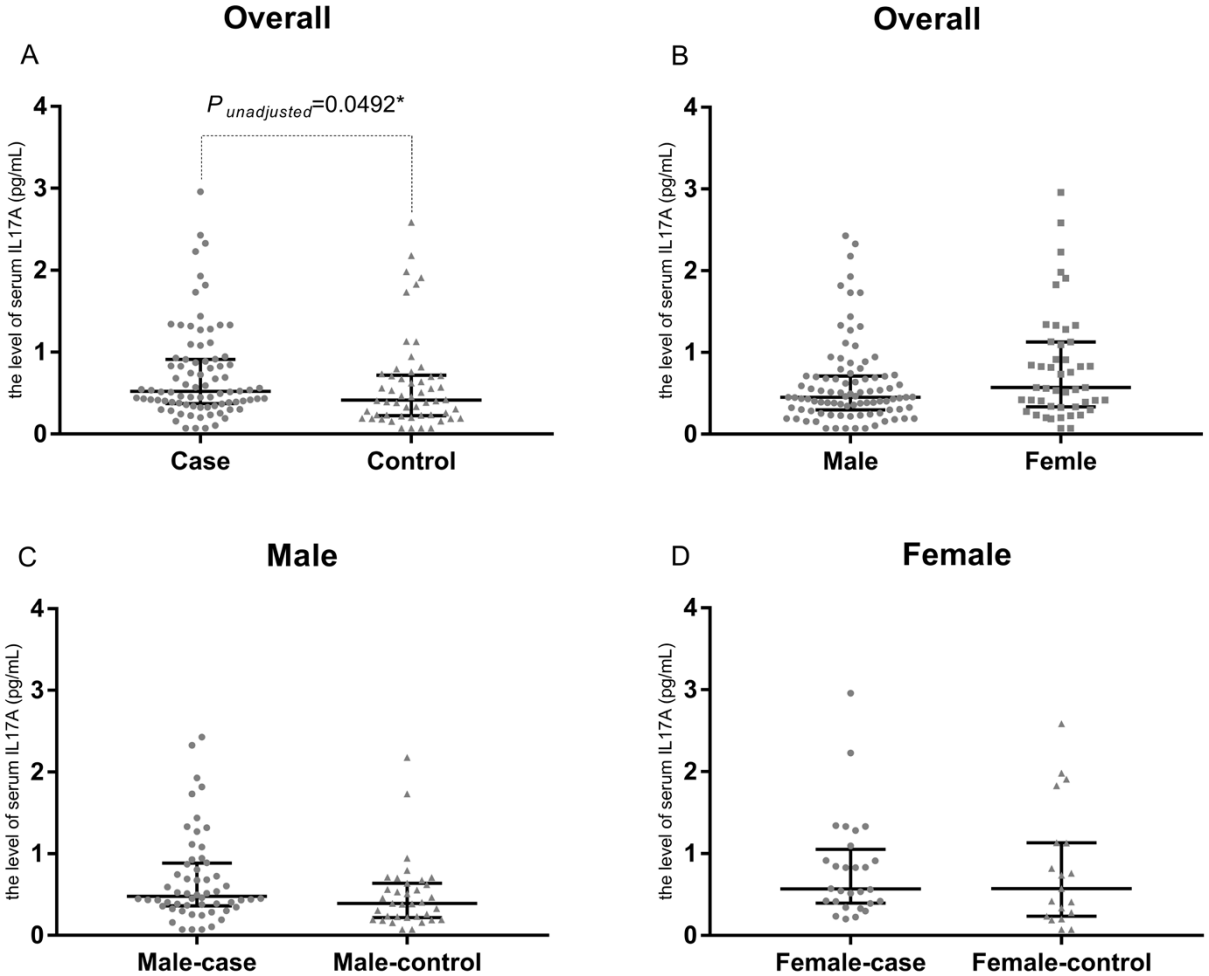
The concentration of serum IL‐17A in DKD case and control groups. (A) Comparison of serum IL‐17A levels between DKD case and control groups in all individuals (84 vs. 54), *P*_*unadjusted*_ = 0.0492; (B) Comparison of serum IL‐17A levels between males and females in all individuals (91 vs. 47), *p* = 0.0703; (C) Comparison of serum IL‐17A levels between DKD case and control groups in male (56 vs. 35), *p* = 0.0606; (D) Comparison of serum IL‐17A levels between DKD case and control groups in female (28 vs. 19), *p* = 0.5156. *p* values were determined by the Mann–Whitney test. The IL‐17A levels are shown as median with interquartile range, respectively.

The level of serum IL‐17A were significantly higher in DKD case group compared with the control group (84 vs. 54, *p* = 0.0496, chisq = 3.8547) (Figure 1A). The median and interquartile range of two groups were 0.520 (0.37, 0.91) and 0.415 (0.23, 0.71), respectively. As we all know, other factors such as sex, age, BMI may also influence DKD, so those confounding factors may be adjusted. After adjustment of sex, age and BMI, the *p* value between groups was 0.0298 (*r* = 0.19). Then we further adjusted sex, age, BMI, the duration of T2DM and HbA1c, and the *p* value was still 0.0439 (*r* = 0.18).

We also stratified those individuals according to sex and tested the difference between case and control groups in the two subgroups. Even the *p* value for difference between case and control in male didn't reach statistic significant, the level of serum IL‐17A in male‐case group tended to be higher than male‐control (56 vs. 35, *p* = 0.0606).

### 3.3 Relationship Between Serum IL‐17A and Renal Function in Patients With T2DM

We mainly used serum creatinine, eGFR and ACR to represent renal function. First, we searched the correlation between the level of serum IL‐17A and the indicators of renal function in patients with T2DM (Table 2). eGFR was significantly associated with the level of serum IL‐17A in unadjusted and adjusted model (*r* = −0.188, *p* = 0.0272; *r* = −0.209, *p* = 0.0167). In unadjusted model, the level of serum IL‐17A just had a slight tendency to be associated with serum creatinine and ACR, but hadn't reached a significance (*p* = 0.1053 and *p* = 0.0500, respectively). After adjusted for sex, age and BMI, the level of serum IL‐17A showed statistic association with serum creatinine and ACR (*r* = 0.213, *p* = 0.0148; *r* = 0.183, *p* = 0.0369).

**TABLE 2:** The correlation of the level of serum IL‐17A with clinical characteristics.

	Unadjusted		Adjusted	
	*r*	*p*	*r*	*p*
Sex	0.155	0.0700	0.141	0.1025
Age	−0.022	0.8015	−0.030	0.7321
BMI	−0.119	0.1637	−0.090	0.2992
Duration of diabetes	−0.003	0.9683	−0.028	0.7492
Serum creatinine	0.138	0.1053	0.213	0.0148*
eGFR	−0.188	0.0272*	−0.209	0.0167*
HbA1c	−0.114	0.1829	−0.135	0.1229
ACR	0.168	0.0500	0.183	0.0369*
Serum uric acid	0.036	0.6757	0.085	0.3354
Blood urea nitrogen	−0.001	0.9881	−0.013	0.8811

*Note:* The correlation of adjusted model for duration of diabetes, serum creatinine, eGFR, HbA1c, ACR, serum uric acid and blood urea nitrogen were adjusted for sex, age and BMI. The correlation of adjusted model for sex was adjusted for age and BMI. The correlation of adjusted model for age was adjusted for sex and BMI. The correlation of adjusted model for BMI was adjusted for sex and age. *p* values < 0.05 are shown in bold.

*p < 0.05.

**FIGURE 2:**
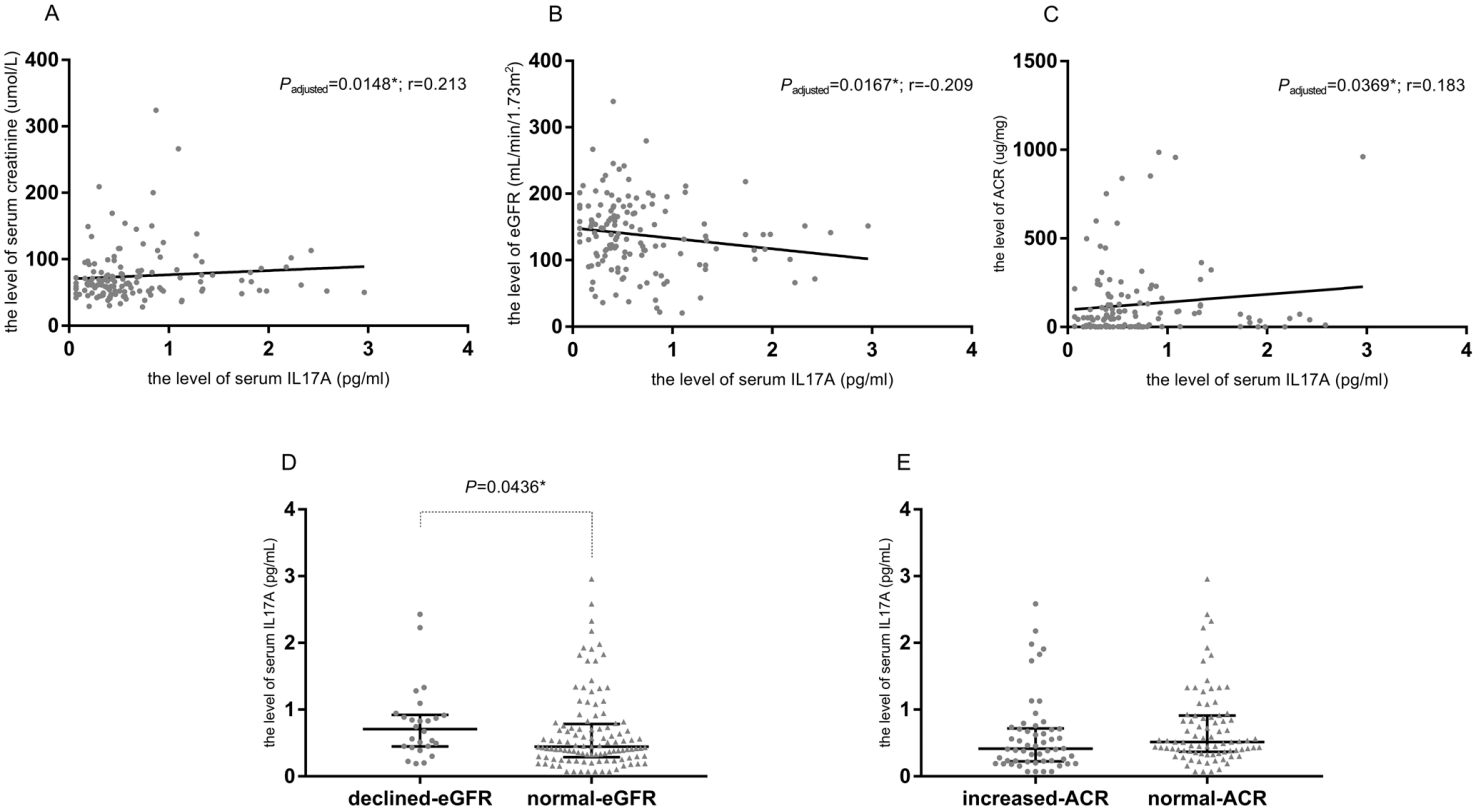
The relationship of the level of serum IL‐17A and the indicators of renal function in patients with T2DM. The relationship of the level of serum IL‐17A with (A) serum creatinine, *p* = 0.0148, *r* = 0.213; (B) eGFR, *p* = 0.0167, *r* = −0.209; (C) ACR, *p* = 0.0369, *r* = 0.183. *p* values were determined by the Spearman correlation. (D) Comparison of serum IL‐17A levels between declined‐eGFR group and normal‐eGFR group (26 vs. 112), *p* = 0.0436; (D) Comparison of serum IL‐17A levels between increased‐ACR group and normal‐ACR group (54 vs. 83), *p* = 0.0588. *p* values were determined by the Mann–Whitney test. The IL‐17A levels are shown as median with interquartile range, respectively.

Because albuminuria was out of sync with eGFR in the progression of DKD, we also carried intergroup comparison according to albuminuria or eGFR. In our population, normal‐eGFR group (eGFR ≥ 90 mL/min/1.73m^2^) had a higher level of serum IL‐17A compared with declined‐ eGFR group (eGFR < 90 mL/min/1.73m^2^) (26 vs. 112, *p* = 0.0436). As to albuminuria, normal‐ACR group (ACR < 30 μg/mg) tended to have a lower level of serum IL‐17A compared with the increased‐ACR group (ACR ≥ 30 μg/mg) (54 vs. 83, *p* = 0.0588). (Figure 2).

### 3.4 Relationship Between Serum IL‐17A and Other Parameters in Patients With Diabetes

We also tested association of other clinical characteristics (sex, age, BMI, duration of T2DM, HbA1c, serum uric acid and blood urea nitrogen) with the level of IL‐17A. Only age had a slight tendency before adjustment (*p* = 0.0700). After adjustment for age and BMI, *p* value for difference between sex was 0.1025. Other parameters didn't show any association with serum IL‐17A. (Table 2).

### 3.5 Patients With Diabetic Retinopathy Had Higher Level of Serum IL‐17A Than Control

**FIGURE 3:**
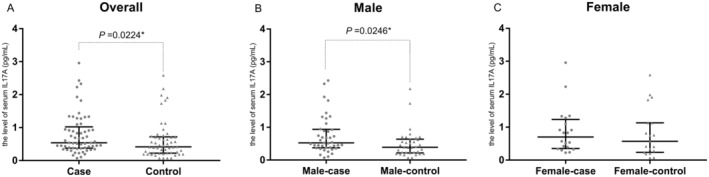
The concentration of serum IL‐17A in DR case and control groups. (A) Comparison of serum IL‐17A levels between DR case and control groups in all individuals (61 vs. 54), *p* = 0.0224; (B) Comparison of serum IL‐17A levels between DR case and control groups in male (41 vs. 35), *p* = 0.0246; (C) Comparison of serum IL‐17A levels between DR case and control groups in female (20 vs. 19), *p* = 0.4482. *p* values were determined by the Mann–Whitney test. The IL‐17A levels are shown as median with interquartile range, respectively.

As we all know, DR is another important diabetic microvascular complication, which may develop with DKD. We also collected the information of DR in our groups. And the control group was also defined as T2DM without DKD or DR. The level of serum IL‐17A was significantly higher in DR case group compared with control group (61 vs. 54, *p* = 0.0224). A similar result was gotten in male, male‐case DR group had higher level of serum IL‐17A than male‐control group (41 vs. 35, *p* = 0.0246). There was no significant difference between case and control in female as the small number of female participants (20 vs. 19, *p* = 0.4482) (Figure 3).

## 4 Discussion

Given the substantial burden of DKD and the limited understanding of its mechanisms, identifying sensitive biomarkers or therapeutic targets is of paramount importance. IL‐17A has been implicated as a key player in DKD pathogenesis, with evidence suggesting a paradoxical role in disease progression [19]. In this study, we investigated the association of serum IL‐17A levels with renal function and diabetic complications in a cohort of 84 DKD patients and 54 T2DM patients without DKD.

Interestingly, despite previous studies emphasising the role of disease duration and glycemic control in DKD development, no significant differences were observed in these parameters between our patient and control groups. This natural balance in traditional risk factors within our sample minimises their potential confounding effects, thereby allowing a clearer evaluation of the association between serum IL‐17A levels and renal function. Our findings revealed that serum IL‐17A levels were significantly higher in the DKD case group compared to the control group (*P*_*unadjusted*_ = 0.0496, *P*_*adjusted*_ = 0.0298), indicating that higher serum IL‐17A came with worse renal function. And serum creatinine, eGFR, ACR were used as indicators of renal function. Serum creatinine and ACR showed positive correlation with the level of serum IL‐17A (r _adjusted_ = 0.213, *P*_*adjusted*_ = 0.0148; r _adjusted_ = 0.183, *P*_*adjusted*_ = 0.0369), while eGFR was negatively correlated with the level of serum IL‐17A (*r*_adjusted_ = −0.209, *P*_*adjusted*_ = 0.0167). These correlations explain the higher serum IL‐17A levels in the DKD group, as declining eGFR and increasing ACR and serum creatinine are established indicators of worsening renal function in DKD patients.

In the progression of DKD, changes in eGFR and albuminuria are known to occur independently [29, 30, 31]. To further investigate the relationship between serum IL‐17A levels and renal function, we compared IL‐17A levels between groups stratified by eGFR and ACR. Consistent with the observed correlations, the declined‐eGFR group exhibited significantly higher serum IL‐17A levels compared to the normal‐eGFR group. Similarly, the increased‐ACR group demonstrated elevated IL‐17A levels relative to the normal‐ACR group.

Other renal function‐related parameters, including serum uric acid and blood urea nitrogen (BUN), did not show a significant relationship with serum IL‐17A in either the adjusted or unadjusted models. This may be partly explained by the fact that serum uric acid and BUN levels can be influenced by dietary factors, and we did not collect dietary information from the participants [32, 33]. Furthermore, in our study, BUN and serum creatinine levels were observed to be lower in the DKD group. This finding could reflect the influence of medications commonly prescribed for DKD patients, such as RAAS inhibitors or diuretics, which can alter these biochemical markers. Additionally, protein‐restricted diets, often recommended for DKD management, may contribute to reduced BUN levels. These factors might obscure a potential relationship between serum IL‐17A and these parameters. Therefore, it is possible that adjusting for diet or medication use could reveal an association between serum IL‐17A and these markers. However, due to the lack of detailed data on these variables, this remains speculative and warrants further investigation.

DR is another common microvascular complication of diabetes, similar to DKD. Evidence suggests a bidirectional relationship between DR and DKD, with each condition representing a risk factor for the other [34, 35]. These findings highlight the interplay between shared and complication‐specific mechanisms that underlie diabetic microvascular complications. To explore this further, we examined the association between serum IL‐17A levels and DR in our cohort. Our analysis revealed that serum IL‐17A levels were significantly higher in the DR group compared to the control group (*p* = 0.0224). This result aligns with previous findings, which reported elevated IL‐17A levels in patients with DR compared to those without this complication [36]. These observations suggest that IL‐17A may contribute to shared pathogenic mechanisms involved in the development of diabetic microvascular complications.

The *p* value for serum IL‐17A levels between male and female participants in our study was approximately 0.1. Although no statistically significant difference was observed, there was a tendency for serum IL‐17A levels to be higher in females. To minimise potential confounding, we stratified the population by sex for further analysis. Among male participants, the results were consistent with those observed in the overall population (*P*_*overall*_ = 0.0492, *P*_*male*_ = 0.0606 for DKD; *P*_*overall*_ = 0.0224, *P*_*male*_ = 0.0246 for DR). However, no statistically significant results were obtained for female participants, likely due to the smaller sample size (28 vs. 19 for DKD; 20 vs. 19 for DR).

Our findings align with previous reports suggesting a role for IL‐17A in DKD pathogenesis, as highlighted in recent reviews [26]. However, unlike prior studies, our analysis specifically focuses on a defined population and evaluates IL‐17A's relationship with renal function after adjusting for traditional risk factors, providing unique insights into its potential role as a biomarker. And accurate range of serum IL‐17A in patients with T2DM was detected as 0.502 (0.306, 0.832) pg/mL. Due to the extremely low serum concentration of IL‐17A (several pg/mL), its detection using conventional ELISA kits has proven challenging. However, advancements in ultra‐sensitive measurement technologies now allow for precise quantification of serum IL‐17A levels. Previous studies have reported varying serum IL‐17A levels across different contexts. For instance, research on the relationship between IL‐17A and oral lichen planus reported serum IL‐17A levels ranging from 8.242–28.663 pg/mL in healthy controls and 8.242–92.282 pg/mL in patients with oral lichen planus [17]. Another study found that serum IL‐17A was 36.1 (15.5–79.1) pg/mL in healthy controls [18]. These reported levels are notably higher than those observed in our study. A key reason for this discrepancy is the higher detection limit of the ELISA kits used in those studies. The ELISA kit we used in this research can detect the IL‐17A concentration range from 0.07 to 60 pg/mL. yielding a median serum IL‐17A level of 0.502 pg/mL. This result aligns closely with findings from more recent studies. [37]. For example, it was reported that the level of serum IL‐17A in patients with systemic sclerosis was 1.36 ± 8.27 pg/mL and in healthy control group was 0.59 ± 1.96 pg/mL [38]. Additionally, the concentration of IL‐17A was 2.9 ± 3.7 pg/mL in metabolism syndrome, while 0.1 ± 0.2 pg/mL in control group [39]. These comparisons highlight the variability in serum IL‐17A measurements across studies and the importance of assay sensitivity in determining accurate concentrations.

This study has several limitations. First, the lack of renal biopsy data, the gold standard for diagnosing DKD, may limit the precision of our findings. Additionally, we did not include urine samples, which could have provided further insight into the expression pattern of IL‐17A and its local involvement in renal pathology. Second, the absence of detailed lifestyle or dietary information could obscure potential confounders, particularly in assessing the relationship between IL‐17A and markers like BUN or serum uric acid. Third, while we identified associations between IL‐17A levels and DR, the lack of data on DR severity precluded a deeper analysis of its relationship with IL‐17A. Nonetheless, our research was the first to prove the relationship of serum IL‐17A and renal function in patients with T2DM. Lager samples size and further research on mechanisms were needed in the future.

## 5 Conclusion

In this study, we employed ultra‐sensitive measurement techniques to evaluate serum IL‐17A levels in individuals with T2DM, with and without DKD, within a Chinese Han population. Our findings revealed that serum IL‐17A levels were significantly higher in DKD patients compared to controls and were associated with indicators of renal function, including positive correlations with serum creatinine and ACR, and a negative correlation with eGFR. Furthermore, we observed a notable elevation of serum IL‐17A levels in patients with diabetic retinopathy, underscoring its potential role in diabetic microvascular complications.

These results suggest that IL‐17A could serve as a promising biomarker for identifying and monitoring DKD and DR progression. Additionally, the findings highlight the potential of IL‐17A as a target for immunotherapy in addressing diabetic microvascular complications. Future studies with larger sample sizes and mechanistic investigations are warranted to validate these findings and explore the therapeutic implications of IL‐17A in diabetes‐related complications.

## Author Contributions

**Li Jin:** conceptualization (equal), data curation (equal), formal analysis (equal), funding acquisition (equal), writing – review and editing (equal). **Yan Huang:** data curation (equal), methodology (equal), writing – original draft (equal). **Wensu Wang:** data curation (equal), formal analysis (equal), investigation (equal), writing – original draft (equal), writing – review and editing (equal). **Jianguo Shen:** writing – review and editing (equal). **Zhuo Chen:** supervision (equal), writing – review and editing (equal).

## Conflicts of Interest

The authors declare no conflicts of interest.

## Data Availability

The data used to support the findings of this study were available from the corresponding author upon request.
